# Efficacy of a Low Dose of Hydrogen Peroxide (Peroxy Ag^+^) for Continuous Treatment of Dental Unit Water Lines: Challenge Test with *Legionella pneumophila* Serogroup 1 in a Simulated Dental Unit Waterline

**DOI:** 10.3390/ijerph13070745

**Published:** 2016-07-22

**Authors:** Savina Ditommaso, Monica Giacomuzzi, Elisa Ricciardi, Carla M. Zotti

**Affiliations:** Department of Public Health and Pediatrics, University of Turin, Turin 10126, Italy; monica.giacomuzzi@unito.it (M.G.); elisa.ricciardi@unito.it (E.R.); carla.zotti@unito.it (C.M.Z.)

**Keywords:** *Legionella*, disinfectants, dental infection control, hydrogen peroxide (H_2_O_2_), biofilm, microbial sensitivity test

## Abstract

This study was designed to examine the in vitro bactericidal activity of hydrogen peroxide against *Legionella*. We tested hydrogen peroxide (Peroxy Ag^+^) at 600 ppm to evaluate *Legionella* survival in a simulated dental treatment water system equipped with Water Hygienization Equipment (W.H.E.) device that was artificially contaminated. When *Legionella pneumophila* serogroup (sg) 1 was exposed to Peroxy Ag^+^ for 60 min we obtained a two decimal log reduction. High antimicrobial efficacy was obtained with extended periods of exposure: four decimal log reduction at 75 min and five decimal log reduction at 15 h of exposure. Involving a simulation device (Peroxy Ag^+^ is flushed into the simulation dental unit waterlines (DUWL)) we obtained an average reduction of 85% of *Legionella* load. The product is effective in reducing the number of *Legionella* cells after 75 min of contact time (99.997%) in the simulator device under test conditions. The Peroxy Ag^+^ treatment is safe for continuous use in the dental water supply system (i.e., it is safe for patient contact), so it could be used as a preventive option, and it may be useful in long-term treatments, alone or coupled with a daily or periodic shock treatment.

## 1. Introduction

Dental units are a reservoir for potential pathogens of human or environmental origin, and dental instruments are believed to be responsible for the transmission of microorganisms by direct contact or by spreading through aerosol sprays created by handpieces (high-speed drills, scalers, air or water syringes). A dental unit is furnished with a system of thin, plastic tubes, called dental unit water lines (DUWLs), which deliver water to the different handpieces, air/water syringes, and ultrasonic scalers.

Contamination can occur when oral organisms [1,2] enter the unit’s water lines through back siphonage when the handpiece is momentarily turned off [3,4]. The entrapped organisms can then be ejected with the water when the piece is turned on again, increasing the potential for cross-infection from patient to patient. To prevent water from passively dripping from the handpieces, air/water syringes, and ultrasonic or Piezo electric scalers, these devices are manufactured with a retraction mechanism. This mechanism can actively “suck-back” contaminants from the oral cavity with the introduction of oral contaminants including microbes into the dental unit waterlines and the dental unit water system. Today, many dental water systems (with retraction mechanisms) are equipped with anti-retraction valves to prevent suck-back of contaminants from the oral cavity and/or are designed to give a short “terminal flush” of water to push out any suck-back [5]. In the in vitro and in vivo experimental studies, even new and unused anti-retraction valves were shown to be quite unreliable, leading to microbial suck-back into the waterline system from the patient [6,7].

Dental units can also become contaminated from the main water supply, which, although potable, still carries bacteria. The first report of contaminated water in DUWLs was published as far back as in 1963 by Blake [8]. Over the last 20 years, many studies [2,9,10] have reported that the primary colonizers of dental-unit water lines are not oral microorganisms, but rather the bacteria that are normally found in potable water (*Moraxella* spp., *Flavobacterium* spp., *Legionella* spp., *Pseudomonas* spp., *Klebsiella pneumoniae*, *Acinetobacter* spp., *Mycobacterium avium*). These organisms colonize and replicate on the inner surfaces of the water line tubing, producing an adherent heterogeneous microbial accumulation called a biofilm. Once formed, the biofilm protects the organisms from desiccation, chemical insult and predation, and it serves as a reservoir that significantly changes the number of free-floating microorganisms in the water exiting the water lines [11,12]. Microorganisms on the surfaces are continuously released from the biofilm into the water that is flowing through or standing in the tube lumen, so that the biofilm becomes the primary reservoir for continued contamination of the system [13,14].

Factors associated with biofilm formation in dental unit water systems could include the following: long periods of stagnation on weekends and evenings, high surface to volume ratio (6:1), nutrients in the water that promote microbial survival, mineral content and hardness of water that promote coating of the lumen, fluid mechanics such as laminar flow, low flow rate, microbial quality (bacteria, fungi, protozoans, and nematodes) of the water entering the system, and failure of anti-retraction valves leading to contamination from the oral cavity of patients [15].

Most of the bacterial species found in DUWLs output water are Gram-negative aerobic heterotrophic environmental bacterial species that exhibit very low pathogenicity, although they may be of concern in the treatment of vulnerable patients, such as immunocompromised and medically compromised individuals [16] and dental staff [17,18]. Nonetheless, there is considerable potential for infection with some bacterial pathogens found in DUWLs output water such as *Pseudomonas aeruginosa*, *Legionella* *pneumophila* and non-tuberculosis *Mycobacterium* species [9,10,17,19,20]. Only a few instances of cross-infection related to DUWLs and associated biofilms have been reported in the literature [21,22]. However, it is still possible that infections caused by DUWLs output water have gone undetected or unreported because of the failure to associate exposure to DUWLs output water and aerosols generated from this water with the development of specific infections. Sporadic infections not requiring hospital admission are also less likely to be investigated. Because of these contaminants, it is important to establish control methods for cleaning and disinfecting the dental water system and for providing quality irrigant/dental treatment water. Different dental chair manufacturers recommend specific products to be used with their equipment. Due to issues of material compatibility, practitioners should consult the manufacturer of their DUWLs prior to introducing any chemical agent, as this may otherwise invalidate their warranty. Depending on the nature of various germicidal agents and the various devices or systems provided with the dental unit, chemical treatment protocols could be used intermittently as a “shock” treatment (higher concentration) and/or continuously introduced into waterlines in small quantities. Although the disinfectants are more efficacious at high concentrations, these levels are limited by the degree of risk to personnel, surfaces or equipment; overall, the continuously applied products performed better than those applied intermittently. This protocol requires having an independent reservoir system that can accommodate the solution of choice.

Peracetic acid [23], ethylenediaminetetraacetic acid tetrasodium salt [24], sodium hypochlorite [25,26], chlorhexidine gluconate [27,28], iodine povidone [28], hydrogen peroxide [29,30] and chlorine dioxide [31,32] are commonly used.

The literature contains several accounts of the properties, germicidal effectiveness, and potential uses for stabilized hydrogen peroxide in health-care settings. Published reports ascribe good germicidal activity to hydrogen peroxide and attest to its bactericidal, virucidal, sporicidal, and fungicidal properties [33,34]. Hydrogen peroxide in different formulations (alkaline peroxide as well as silver added to H_2_O_2_) has been studied with respect to biofilm control and long-term treatment [35,36] because the product is safe for patient contact.

This study was designed (1) to examine the in vitro bactericidal activity of hydrogen peroxide against *Legionella pneumophila* serogroup (sg) 1 at 600 ppm and (2) to evaluate *Legionella* *pneumophila* sg 1 survival in simulated dental treatment water line equipped with Water Hygienization Equipment (W.H.E.) device that is artificially contaminated and treated continuously with 600 ppm of hydrogen peroxide with silver ions that boost the biocide action.

## 2. Methods

All experimental protocols were performed with two replicates, and they were repeated on at least two separate occasions. Although *Legionella* are hazard group 2 organisms, all experimental tests were performed in a microbiological safety cabinet according to normal laboratory safety procedures.

The 3% Peroxy Ag^+^ was diluted to a final concentration of 600 ppm. As hydrogen peroxide at high concentration has strong oxidizing properties, it is responsible for adverse effects on dental chair unit components. We wanted to determine a much lower and safer concentration of Peroxy Ag^+^ in municipal water as irrigant/coolant for use in dental treatment. At this concentration Peroxy Ag^+^ can be continuously applied in DUWLs.

### 2.1. Antimicrobial Efficacy of Peroxy Ag^+^: In Vitro Tests 

The efficacy of diluted Peroxy Ag^+^ (a H_2_O_2_ 3% based disinfectant with 0.001% Ag^+^, produced by Cefla S.C., Imola, Italy) was tested according to the method described in UNI EN 13623 “Quantitative suspension test for the evaluation of bactericidal activity against *Legionella* of chemical disinfectants for aqueous systems” [37]. This method is suitable for products used in water for general purpose like spas, showers, and others uses. It specifies the minimum requirements for bactericidal activity of chemical disinfectant products intended to be used for treatment in aqueous systems against *Legionella pneumophila* sg 1. It is assumed that the product has antibacterial properties if it causes a minimum 4 log reduction in the number of viable bacteria after 60 min, when the test organisms is *Legionella pneumophila* sg 1 ATCC 33152. Anti-*Legionella* activity was also studied after 15 h of contact time, as it is the longest time specified by the standard 13623 for slower acting products. In order to take into account the intended specific usage conditions (dental setting), additional specific bactericidal activity was determined by testing contact times of 10 min and 75 min.

Briefly, Peroxy Ag^+^ was diluted to 600 ppm in hard water and added to a test suspension (N_0_) of *Legionella pneumophila* sg 1 at a concentration of 1 × 10^7^ cells/mL in a solution of interfering substance. The mixture was maintained at 20 °C for intended contact time. At the end of the contact time, an aliquot was taken and neutralized with catalase. After 5 min ± 10 s of neutralization time, a 1 mL sample of the neutralized test mixture was immediately taken in duplicate and inoculated on BCYEα agar (Oxoid, Wesel, Germany) using spread-plate technique. The plates were incubated at 36 ± 1 °C in ambient air. According to the general instruction for validation and control procedures of EN13623, the absence of toxicity of the neutralizer was verified, and the dilution-neutralization tests were validated at the same time under the same conditions.

All plates were checked for positive or negative growth after seven days. The number of surviving bacteria (N_a_) in each sample was determined and the reduction was calculated. Mean CFU/mL was converted into a decimal log value for normalization, and decimal log reduction (lgR) was calculated using the following equation:

lgR = lgN_0_ − lgN_a_ (1)

### 2.2. Antimicrobial Efficacy of Peroxy Ag^+^: Challenge Tests 

To assess the efficacy of Peroxy Ag^+^ in continuous exposure, a dental unit water system simulation device was used, with a length of approximately five meters of waterlines. The system was equipped with one handpiece (air/water syringe) and supplied with a dosing device W.H.E., which was specifically designed to operate with continuous treatment of incoming municipal water entering the dental unit by an automatic addition of Peroxy Ag^+^ (approximately 600 ppm as H_2_O_2_) in order to deliver the procedural water to the dental instruments and to the glass filler for mouth rinsing.

The same disinfectant can also be used at higher concentration (3% for H_2_O_2_) for discontinuous daily shock treatments by means of a supplementary expansion of the device, provided by the same manufacturer, which has not been tested in this study.

**Figure 1:**
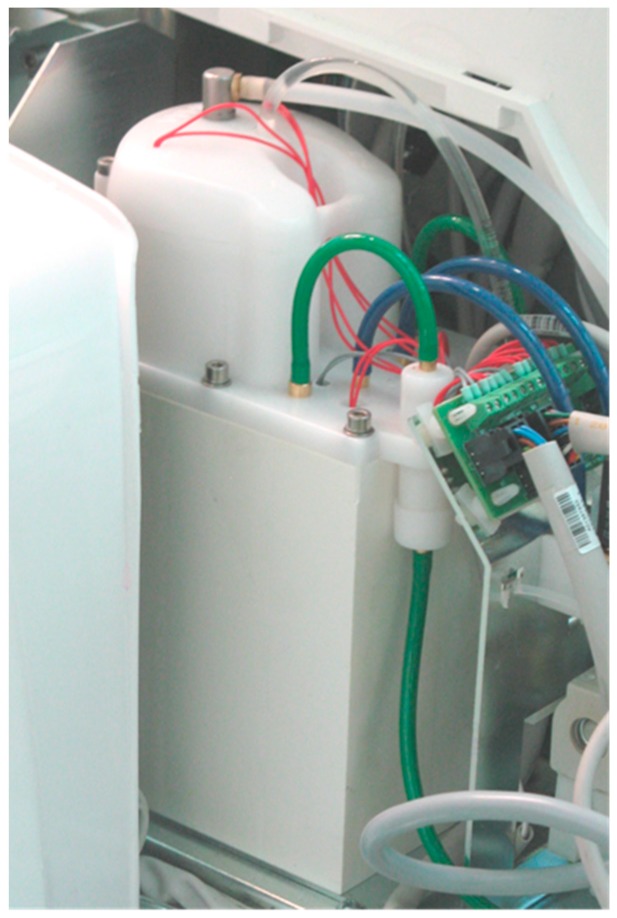
The Water Hygienization Equipment (W.H.E.) device inside a dental unit, at the inlet of the municipal water.

Both the continuous device and the discontinuous system can be provided as built-in systems in new dental units or later integrated in many models of the same manufacturer (Figure 1).

The H_2_O_2_-based disinfectant was used to meet exigencies of the user who require an effective but non-hazardous product that is environmental friendly (H_2_O_2_ spontaneously decomposes into oxygen and water) and compatible with the materials of the dental unit waterlines.

**Figure 2:**
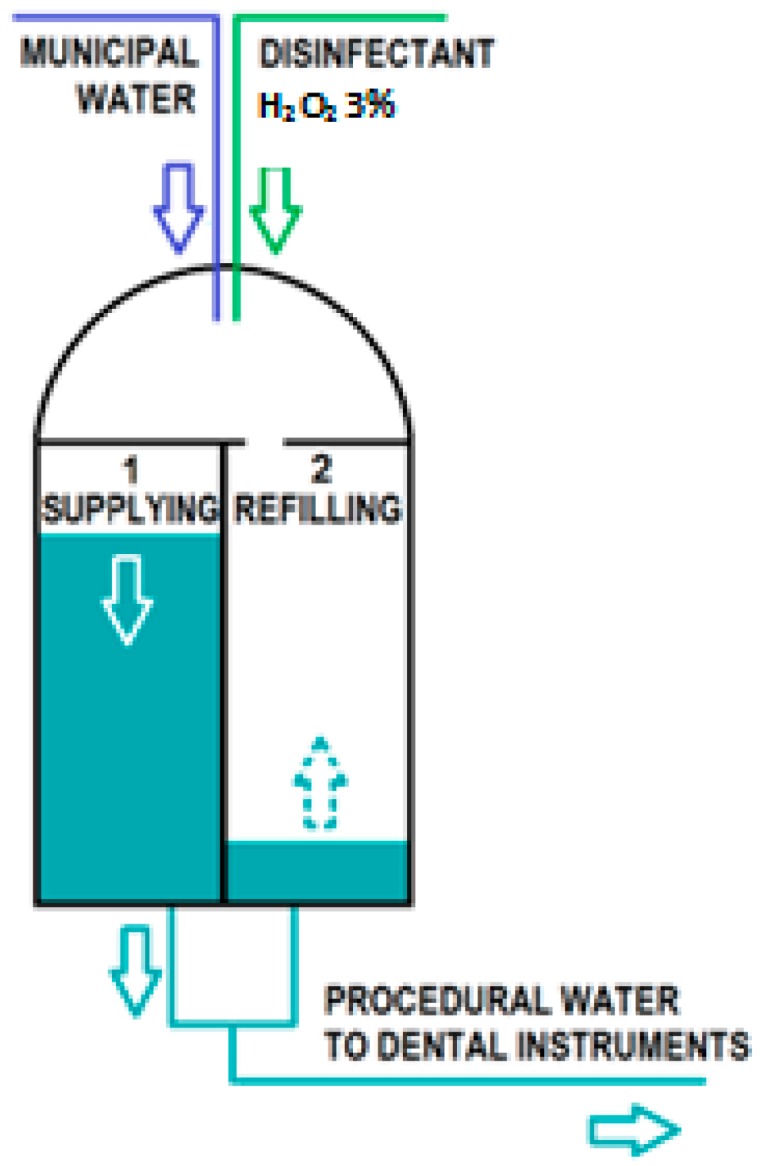
Technical structure and operation of the W.H.E. device.

**Figure 3:**
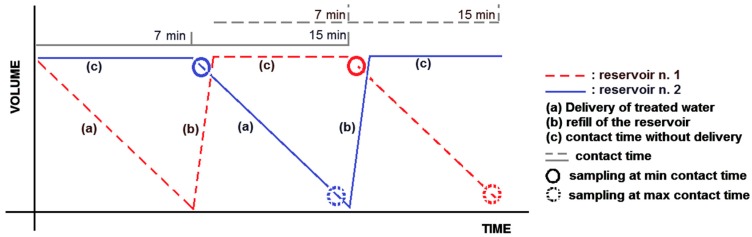
Delivery of treated water from the W.H.E. device.

To perform the continuous treatment, the W.H.E. device uses two “supply reservoirs”, 1 and 2, containing approximately 240 mL each, which alternately supply treated procedural water to the dental instruments (Figure 2 and Figure 3).

As shown in Figure 2, reservoir 1 starts to deliver treated water (in Figure 3: dashed line tract (a)). When reservoir 1 is empty, a refilling phase starts (dashed line tract (b)), in which municipal water and approximately 4 mL of Peroxy Ag^+^ from a common upper chamber is supplied to reservoir 1. After a few seconds, reservoir 1 becomes full with a 600 ppm solution of H_2_O_2_, and a “contact phase” begins (dashed line tract (c)) without any delivery of treated water from reservoir 1. At the same time, reservoir 2 starts working (continuous line tract (a)) and supplies treated water in the place of reservoir 1 (Figure 2). After a few minutes—approximately 7–15 min depending on the flow and the activation time of the procedural water—reservoir 2 also becomes empty, and it begins its refilling phase (continuous line tract (b)). The supplying function shifts to reservoir 1, where the municipal water has already been in contact with the disinfectant.

The W.H.E. device used in this study was isolated from the dental unit, and it was supplied with water coming from an independent pressurized bottle in place of the main water and was operated with a continuous flow of 30–35 mL/min of delivered treated water. This simulated continuous consumption of procedural water by dental instruments in a dental unit.

Prior to use, a dedicated reservoir was filled with Peroxy Ag^+^, solution and the disinfectant reservoir was connected to the W.H.E. device. The pressurized bottle, simulating the incoming municipal water*,* was contaminated with *Legionella pneumophila* sg 1 ATCC 33152.

The inoculum of *Legionella* was obtained after subculturing for three days on BCYEα media at 36 ± 1 °C in ambient air. On the day of the test, bacteria were harvested by swabbing the surface of the agar and were dispersed in 10 mL of Page’s saline solution. A suspension (N_0_) of the challenge microorganism (adjusted with Page’s saline to contain approximately 1 × 10^7^ cells/mL) was placed in self-contained water reservoirs (1.8 L pressurized bottle). The bottle was placed on a shaker, and 10 mL aliquots were sampled. The aliquots were serially diluted tenfold and spread-plated on BCYEα agar in order to confirm the *Legionella* concentration in the bottle. The pressurized bottle was connected to the simulator, and water delivery was started.

#### 2.2.1. Collection of Water Samples

The treated water (*Legionella* cells and 600 ppm of Peroxy Ag^+^) supplied by the W.H.E device was delivered and collected. The effluent was sampled six times from the handpiece, both at the beginning and at the end of the delivering phase (contact time: 7 min and 15 min; delivery time from W.H.E. reservoirs to handpiece output: 1 min 30 s) from each reservoir (Figure 3). To take into account the stagnation of the water when the DUWLs were at rest, we collected water after a hold time of 60 min (75 min of max contact time).

#### 2.2.2. Laboratory Procedure

The effluent was neutralized with catalase and a 1.0 mL aliquot of the neutralized samples was serially diluted tenfold and spread-plated on BCYEα agar, and then incubated at 36 ± 1 °C in ambient air for seven days before colonies were counted (N_a_). The mean CFU/mL was converted into decimal log values for normalization of the data, and decimal log reduction (lgR) was calculated using the Equation (1).

## 3. Results 

### 3.1. In Vitro Test

When *Legionella pneumophila* sg 1 was exposed to the product for 10 min, the effectiveness of the product was not demonstrated. When treatment was prolonged according the obligatory test conditions (contact time 60 min), we obtained a two decimal log reduction. High antimicrobial efficacy was obtained with extended periods of exposure: four decimal log reduction at 75 min and five decimal log reduction at 15 h of exposure (Table 1).

### 3.2. Challenge Test 

#### 3.2.1. Continuous Mode

The results of the challenge test are shown in Table 2.

At the start, the final suspension produced a challenge concentration of 5.70 × 10^3^ cells/mL of *Legionella pneumophila* sg 1. At 8 min 30 s of exposure time, Peroxy Ag^+^ showed a 0.95 lgR corresponding to an 88.77% kill rate of bacterial counts, and at 16 min 30 s of exposure time, Peroxy Ag^+^ showed a 0.75 lgR corresponding to an 82.21% kill rate.

#### 3.2.2. At Rest Stagnation

The results of the challenge test are shown in Table 2. At the start, the effluent water from the line had a concentration of 5.0 × 10^4^ cells/mL. After a one-hour exposure (at rest), no organisms were recovered, and Peroxy Ag^+^ showed a 4.69 log reduction, corresponding to a 99.997% bacterial kill rate.

## 4. Discussion

Dental patients and dental staff can be exposed to pathogenic microorganisms including cytomegalovirus (CMV), hepatitis B virus (HBV), hepatitis C virus (HCV), herpes simplex virus types 1 and 2, HIV, *Mycobacterium tuberculosis*, staphylococci, streptococci, and other viruses and bacteria that colonize or infect the oral cavity and respiratory tract.

These organisms can be transmitted in dental settings through (1) direct contact with blood, oral fluids, or other patient materials; (2) contact of conjunctival, nasal, or oral mucosa with droplets containing microorganisms generated from an infected person and propelled a short distance; (3) inhalation of airborne microorganisms that can remain suspended in the air for long periods; and (4) indirect contact with contaminated objects (e.g., instruments, equipment, DUWL, or environmental surfaces) [38].

Appropriate procedures to decontaminate handpieces, including autoclaving and handpiece replacement between patients, have been developed and implemented in dental practices. These procedures are aimed at reducing the likelihood of aerosol dissemination of pathogens within dental operatories, which can lead to infections. However, decontamination of handpieces (such as high-speed drills and syringes) does not eliminate potential exposures to pathogens that originate from the waterlines of dental units [39].

Various products have been developed to treat the water used in DUWLs so as to reduce the number of bacteria delivered to the patient.

Because disinfectants for registration are tested in vitro and the construction characteristics of the dental units vary widely, the field tests and disinfection protocols are tailored to the individual devices. It is not usual to find a disinfection protocol that is effective for all. This study focused on the bactericidal activity of a low dose of hydrogen peroxide at 600 ppm on *Legionella* *pneumophila* sg 1. The availability of the simulation device allowed us to test both the continuous disinfection of the incoming water for dental treatment proposed by the manufacturer and the residual disinfecting effect on stagnant water inside the waterlines downstream of the device itself. The artificially contaminated simulation device made it possible to measure, under controlled conditions, the performance of the continuous treatment operated by the W.H.E. device towards *Legionella*, in the absence of variables such as biofilms, flow intensity, other microbial species, temperature, and chemical characteristics of the water.

The effects of a discontinuous and stronger disinfecting system (shock treatment) proposed by the same manufacturer for a daily disinfection of waterlines with Peroxy Ag^+^ 3%, as specified above, were not analysed in this study.

In a first set of experiments, the effectiveness of the product was demonstrated at the required log reduction only after long periods of exposure: Peroxy Ag^+^ achieved a four decimal log reduction at 75 min (99.993%) and a five decimal log reduction (99.9995%) after overnight treatment (15 h). This last contact time reflects a realistic period of inactivity in which the chemical product (although at low doses) could prevent the proliferation of bacteria.

In a second set of experiments involving a simulation device, the results showed that when Peroxy Ag^+^ is flushed into the simulation DUWL (contact time: 8 min 30 s–16 min 30 s), we obtained an average reduction of 85% of *Legionella* load. The product is effective in reducing the number of *Legionella* after 75 min of contact time (99.997%) in the simulator device under test conditions. The differences in bactericidal activity between the tests conducted in vitro at 10 min and the challenge test at 8 min and 30 s were probably due to the difference in concentration of *Legionella* *pneumophila* sg 1 used to contaminate the device (5.7 × 10^3^/mL). The concentrations used in vitro were required to be in the range of 1.5 × 10^7^ to 5.0 × 10^7^/mL. Perhaps the high concentrations of cells and the subsequent production of catalase caused these differences in results.

When hydrogen peroxide is used, it is important to consider that it produces destructive hydroxyl free radicals that can attack membrane lipids, DNA, and other essential cell components. Catalase, produced by aerobic organisms and facultative anaerobes with cytochrome systems, can protect cells from metabolically produced hydrogen peroxide by degrading it to water and oxygen. *Legionella* also produces catalase to deactivate peroxide radicals, allowing them to survive in the host cell. According to Bandyopadhyay [40], two genes, *katA* and *katB*, have been correlated with aerobic growth and H_2_O_2_ resistance and replication in macrophages. This defense is overwhelmed by the concentrations used for disinfection. Organisms with high cellular catalase activity (e.g., *Staphylococcus aureus*, *Serratia marcescens*, and *Proteus mirabilis*) required 30–60 min of exposure to 0.6% hydrogen peroxide for a 10^8^ reduction in cell counts, whereas organisms with lower catalase activity (e.g., *Escherichia coli*, S*treptococcus* spp., and *Pseudomonas* spp.) required only a 15 min exposure [33].

The results obtained in this “ideal” situation may represent a limitation of this study because the absence of a biofilm facilitates contact and increases the effectiveness of the product under evaluation. These results may represent what could be accomplished by installing the device on the dental unit in which there is no biofilm (new dental unit) or in which the biofilm was removed by shock treatments. The “*Legionella* free” condition could be maintained by means of continuous disinfection in the latter case.

## 5. Conclusions

The Peroxy Ag^+^ treatment is safe for patient contact, so it could be used as a preventive option, and it may be useful in long-term treatments, alone or coupled with a daily or periodic shock treatment.

Because previous studies have shown that aerobic heterotrophic bacteria are the predominant organisms present in DUWLs [11,41,42,43,44], this study could offer a basic tool to assess the efficacy of W.H.E. device + Peroxy Ag^+^ in dental practices by monitoring the total heterotrophic bacteria contamination in DUWLs.

## Figures and Tables

**Table 1:** Kill rate of *Legionella pneumophila* serogroup (sg) 1 exposed to 600 ppm Peroxy Ag^+^ according to European Standard 13623 (in vitro test).

Time of Exposure	Inoculum (N_0_)	Outcome (N_a_)	lgR	Kill Rate %
10 min	4.89 × 10^7^ (7.69)	3.47 × 10^7^ (7.54)	0.15	29.20
60 min	4.89 × 10^7^ (7.69)	1.95 × 10^5^ (5.29)	2.40	99.60
75 min	4.89 × 10^7^ (7.69)	3.16 × 10^3^ (3.50)	4.19	99.993
15 h	4.89 × 10^7^ (7.69)	2.1 × 10^2^ (2.30)	5.39	99.9995

lgR = lgN_0_ − lgN_a_; N_0_: the number of cells per mL in the test mixture; N_a_: the number of surviving cells per mL in the test mixture at the end of exposure; R: viable cell reduction factor.

**Table 2:** Kill rate of *Legionella pneumophila* sg 1 exposed to 600 ppm Peroxy Ag^+^ in the simulation device.

Time of Exposure	Inoculum (N_0_)	Outcome (N_a_)	lgR	Kill Rate %
8 min 30 s *	5.70 × 10^3^ (3.75)	6.33 × 10^2^ (2.80)	0.95	88.77
16 min 30 s *	5.70 × 10^3^ (3.75)	1.02 × 10^3^ (3.00)	0.75	82.21
75 min **	5.00 × 10^4^ (4.69)	0	≥4.69	99.997

* contact times in reservoirs: 7 min; 15 min; delivery time from W.H.E. reservoirs to handpiece output: 1 min 30 s; ** contact time in reservoirs: 15 min; hold time: 60 min; lgR = lgN_0_ − lgN_a_; N_0_: the number of cells per mL in the test mixture; N_a_: the number of surviving cells per mL in the test mixture at the end of exposure; R: viable cell reduction factor.
